# Investigating fecal microbial transplant as a novel therapy in dogs with inflammatory bowel disease: A preliminary study

**DOI:** 10.1371/journal.pone.0276295

**Published:** 2022-10-18

**Authors:** Allison J. Collier, Diego E. Gomez, Gabrielle Monteith, Brandon L. Plattner, Adronie Verbrugghe, Jinelle Webb, J. Scott Weese, Shauna L. Blois

**Affiliations:** 1 Department of Clinical Studies, Ontario Veterinary College, Guelph, Ontario, Canada; 2 Department of Population Medicine, Ontario Veterinary College, Guelph, Ontario, Canada; 3 Department of Diagnostic Medicine and Pathobiology, Kansas State University, Manhattan, Kansas, United States of America; 4 Mississauga Oakville Veterinary Emergency Hospital, Mississauga, Ontario, Canada; Thomas Jefferson University, UNITED STATES

## Abstract

**Background:**

There are limited studies investigating the use of fecal microbial transplant (FMT) in dogs with inflammatory bowel disease (IBD). The aim of this preliminary study was to assess the feasibility of adding FMT to standard therapy (corticosteroids and a hypoallergenic diet) for dogs with IBD and to and to describe the changes in measured outcomes after 30 days of treatment.

**Methods:**

Thirteen client-owned dogs with IBD were enrolled in this double blinded, randomized clinical trial. All dogs received corticosteroid therapy and a hypoallergenic diet; dogs were randomized to receive either placebo or FMT. Measured outcomes included the canine chronic enteropathy clinical activity index (CCECAI) at 1 week and 1 month after enrolment. Fecal microbiota were analyzed after extracting DNA from fecal samples and profiling using 16S amplicon sequencing. Dogs in the placebo group not responding to treatment after 1 month were offered FMT.

**Results:**

The CCECAI significantly decreased over time in both groups (p = 0.001). There were no significant differences between the CCECAI of the placebo and FMT group at each time point (F test from ANOVA, p = 0.40). No adverse effects were reported in the 30 days following FMT.

**Conclusions:**

The addition of FMT to standard therapy for IBD was feasible. No significant differences were observed in the CCECAI between groups at each time point. Large scale clinical trials can be performed using these methods to evaluate the longer term effect of FMT on clinical signs, microbial diversity, and other outcomes.

## Introduction

Inflammatory bowel disease is one of the most frequent causes of chronic vomiting, anorexia and diarrhea in dogs [1,2]. The gastrointestinal microbiota has been speculated to be an integral component of the pathogenesis of IBD. Dysbiosis has been noted in both humans and dogs with IBD [3–6].

There is a lack of consensus on the most appropriate treatment for dogs with IBD. Empirical treatment is largely employed, with various antimicrobial, dietary, and immunomodulatory therapies [1]. Despite treatment, a considerable number of patients continue to have clinical signs. As a result, additional treatment modalities to improve the response for dogs with IBD have been explored. Fecal microbial transplantation (FMT) has been investigated in human medicine for treatment of various gastrointestinal diseases and could be applicable in veterinary medicine [7–10].

Fecal microbial transplantation has been beneficial in people with refractory *Clostridioides difficile* infection, with mean cure rates of up to 90% after FMT [11–14]. More recently, it has been used utilized in people with IBD to treat the dysbiosis suspected to contribute to disease pathogenesis [15–17]. Fecal microbial transplantation has resulted in clinical improvement of dogs with IBD in several case reports and series, although further clinical trials are necessary to understand the efficacy of FMT in dogs with IBD [18,19].

The primary objective of this study was to examine feasibility of adding FMT to the treatment of dogs diagnosed with IBD and treated with standard treatment (i.e., hypoallergenic diet and immunosuppressive medications). The second objective was to describe the outcome of treatment with either standard treatment plus FMT versus standard treatment plus placebo after 30 days of treatment. Additionally, a goal of this preliminary study was to assess for potential adverse effects of FMT after 30 days, as well as to gather information to aid in planning larger, long-term studies on FMT in dogs.

## Materials and methods

### Study population

In this randomized, parallel, double-arm, single-centre clinical trial, a convenience sample of client owned dogs with IBD were recruited from the Ontario Veterinary College Health Sciences Centre between September 2018 and August 2020. All dogs were privately owned, the owners signed an informed consent, and the study was approved by the Institutional Animal Care Committee.

Dogs were eligible for inclusion if they had a greater than 3-week history of clinical signs consistent with IBD, as defined by the CCECAI [20], and histopathologic evidence of lymphocytic, plasmacytic, and/or eosinophilic IBD on histopathology of the upper and/or lower gastrointestinal tract. Non-gastrointestinal causes of the clinical signs were investigated for with a complete blood count (CBC), serum biochemistry, fecal flotation parasite testing, serum cobalamin testing, and abdominal ultrasound in all dogs. Additional diagnostic tests including resting serum cortisol levels were performed at the discretion of the primary clinician. Enrolled dogs were free of antimicrobial treatment for at least 1-week prior to enrollment and had not received corticosteroids for >2 weeks prior to enrolment.

Animals were excluded from enrollment if any evidence of primary lymphangiectasia was present in the gastrointestinal tract histologically or if there was evidence of comorbidities that could cause clinical signs of chronic enteropathy. Breeds predisposed to *E*.*coli*-related histiocytic ulcerative colitis (e.g., Boxer, French Bulldog) were excluded from the study.

### Fecal microbial transplant preparation

Ten healthy canine fecal donors were recruited from the Guelph community (Guelph, Ontario, Canada) throughout the study period. Fecal donors were deemed to be of appropriate health status based on normal physical examination, CBC and serum biochemistry within the preceding 3 months. Fecal donors had no history of vomiting or diarrhea in the past 6 months, skin disease, exposure to raw food diets, antimicrobial use in the previous 6 months, or major medical conditions including bacterial infections. Donor fecal samples were negative for parasites via fecal flotation and *Giardia* ELISA, and negative for *Salmonella*, *Clostridium difficile*, *and Campylobacter spp*. on fecal culture. Fecal samples were collected as voided and frozen at -20°C within 24 hours of collection for up to 3 months for later FMT preparation.

For preparation of FMT aliquots, fecal samples were selected from five donors and pooled to decrease the effect of individual donors on treatment outcome. Feces were thawed at room temperature for 2 hours prior to preparation and then 10g of feces from five donors were obtained (50g total). Fecal material was blended with sterile saline at a ratio of 1 part feces to 5 parts sterile saline. This solution was sieve filtered then stored in 60mL syringes at -20°C until usage. Fecal microbial transplant preparations and fecal donor samples were discarded after 3 months and donor samples underwent no more than one freeze-thaw cycle prior to FMT preparation. Fecal donors were retested yearly as described above. Additional screening for extended-spectrum beta-lactamase (ESBL)-producing *Escherichia coli (E*. *coli)* was initiated in October 2019 and performed throughout the remainder of the study.

### Study design and fecal microbial transplant administration

Enrolled dogs were randomized using an online randomization program (https://www.sealedenvelope.com) into either the FMT or the placebo group. All dogs were prescribed standard therapy for IBD, comprised of immunosuppressive doses of prednisone (approximately 2mg/kg/day) and a hypoallergenic (hydrolyzed or novel protein) diet. Fecal microbiota transplant or placebo was administered within 2 weeks of initiating standard therapy.

All investigators except one (SLB) remained blinded to group allocation throughout the study. Following randomization, dogs were admitted into the hospital to undergo infusion of either the FMT preparation or saline via retention enema by the unblinded investigator. Aliquots of FMT were thawed at room temperature for approximately 1-hour prior to administration. Placebo was a similar volume of sterile saline at room temperature. The FMT or placebo enema was administered via a sterile lubricated Red Rusch tubing in the descending colon over 1–5 minutes. A total infusion volume of 10mL/kg of FMT or sterile saline was administered and retained within the colon for at least 10 minutes following infusion. Gauze was inserted in the rectum in dogs, if needed, to facilitate retention. Dogs were discharged the same day following the procedure. Early in the study, the FMT or placebo enema was administered within 2 weeks of endoscopy after confirmation of a diagnosis of IBD. Due to challenges retaining dogs in the study with this approach, beginning in January 2020 enemas were administered during general anesthesia following endoscopy in dogs where the clinician had a high suspicion of IBD. Any dog receiving FMT or placebo immediately following endoscopy that ultimately did not have histologic evidence of IBD was excluded from further study visits or any data analysis.

Enrolled dogs were subsequently evaluated at 1 week and then at 1 month following FMT or placebo treatment. At each evaluation, the clinical status was scored by a blinded investigator (AJC), utilizing the CCECAI. Feces were obtained within 12 hours of each visit either following natural voiding or via digital rectal examination and stored at -80°C until further analysis. At each recheck, serum biochemistry or other diagnostics were performed at the discretion of the attending clinician, based on the dog’s clinical status and in consultation with the client. Immunomodulatory and other therapies were adjusted at each evaluation based on patient status at the discretion of the primary clinician.

Dogs not responding to treatment (based on lack of improvement in the CCECAI and/or owner perception of clinical signs) were offered to have their treatment groups revealed 3 months after enrolment. Beginning in November 2019, it was elected to shorten the time period of unblinding to 1 month following treatment to facilitate earlier FMT or other interventions for dogs having previously received a placebo if no significant response was noted. Owners of dogs in the placebo group were given the option of having FMT administration via retention enema as previously described and were then reevaluated 1 week and 1 month following FMT. These dogs were labeled as FMT2 and were included only in specified analysis below. Responders were continued to have their treatment group blinded to the owner and study investigator performing patient assessment.

### Blood analyses

Serum biochemistry (Cobas 6000 c5-1, Roche Diagnostics, Hillsdale, MI, USA), CBC (Advia 2120 hematology analyzer, Siemens Health Diagnostics, Tarrytown, NY, USA), or other diagnostics unless otherwise specified were performed at the Animal Health Laboratory at the Ontario Veterinary College.

### Fecal DNA extraction and PCR

Prior to deoxyribonucleic acid (DNA) extraction, fecal samples were brought to room temperature for 1–2 hours. Fecal samples underwent DNA extraction using the E.Z.N.A. Stool DNA Kit Pathogen Detection Protocol (Omega Bio-Tek Inc., Doraville, Georgia, USA), performed as per the manufacturer’s instructions. DNA samples were then stored at -20C until polymerase chain reaction (PCR).

Following this, the 16S rRNA genes were amplified through targeting the V4 region. The PCR reaction mixture contained 12.5μL of Kapa HiFi Ready Mix (Kapa Biosystems, Wilmington, Massachusetts, USA), 9.5μL of nuclease-free water, 2μL of DNA and 0.5μL of forward (S-D-Bact-00564-a-S-15 ′′′′ 5 -AYTGGGYDTAAAGNG-3) and reverse (S-D-Bact-0785-b-A-18 5-TACNVGGGTATCTAATCC-3) primers (10 pMol/μL) [21]. A molecular grade water sample was used as a negative control during this stage.

The PCR products were then purified with magnetic beads and then were amplified by PCR with Illumina adapters (Mastercycler Pro, Eppendorf Canada Ltd., Mississauga, Ontario, Canada). They were then purified a second time. The NanoDrop® (NanoDrop 1000 Spectrophotometer, Nano Drop Technologies Inc. (Thermo Fisher Scientific), Waltham, Massachusetts, USA) was used to quantify DNA through spectrophotometry [22]. Gel electrophoresis was utilized to evaluate the final PCR products. The library was pooled and sequencing was performed at the University of Guelph’s Advanced Analysis Centre, using an Illumina MiSeq platform (Illumina, San Diego, California, USA). For a positive control, a mixed microbial community was utilized (20 Strain Even Mix Genomic Material, MSA-1002, ATCC (Manassas, Virginia, USA) distributed from Cedarlane Laboratories (Burlington, Ontario, Canada)).

### Sequence processing and data analysis

Data analysis was carried using the software Mothur v.1.39.5 [23], through a previously published protocol [24]. After assembly of paired end reads through the make.contigs command, sequences greater than 292 base pairs were removed. Good quality sequences were aligned against the SILVA database, with removal of sequences that did not align to the correct region [25]. Chimeras were identified and removed [26]. Taxonomic assignment of sequences was performed through the Ribosomal Database Project classifier (v14) and archaea were removed [27]. Based on the smallest number of sequences from a sample, subsampling was performed.

### Statistical analysis

For all statistical analyses, significance was set at p <0.05. Data were checked for normality with the Shapiro Wilk test. Non-normal data were log transformed to meet the assumptions of normality.

A general linear model that accounted for the repeated effect of measuring the same dog over time was used to test for differences in the CCECAI between the placebo and the FMT group. Examination of the residuals assessed the data distribution and checked for possible outliers. Data for the CCECAI were normally distributed and there were no outliers. Fixed effects included in the model were group and day, as well as their interaction. Post hoc Dunnett’s tests were applied to compare the effect of day back to baseline.

For statistical analyses, dogs in the placebo group that subsequently received FMT were labelled as ‘FMT2’ for each timepoint from when they received FMT onwards. The FMT2 group was excluded from further analysis except where explicitly indicated.

### The gastrointestinal microbiota

Alpha diversity was assessed using the Chao-1 index, Inverse Simpson, and Shannon Evenness indices. Statistical analysis of alpha diversity was performed using JMP 15.2 (SAS Campus Drive, Cary, North Carolina, USA). Beta diversity was assessed using the Yue and Clayton index to assess community structure, and Jaccard index to assess community composition.

To assess for significant differences between groups in beta diversity, the analysis of molecular variance (AMOVA) was utilized in Mothur [23]. Statistical differences in the relative abundances between disease groups were investigated using the linear discriminant analysis effect size (LEfSe) using a Linear Discriminant Analysis (LDA) cut-off value >2 and p<0.05.

## Results

### Study population

Thirteen dogs with clinically and histologically confirmed IBD were prospectively enrolled. Dogs were randomly assigned into the placebo group (n = 6) or the FMT group (n = 7). Age at presentation ranged from 1–11 years of age. Breeds represented included a Yorkshire terrier (2), German shepherd (3), mixed breed (6), pug (1), and Labrador retriever (1). Ten dogs were female, and three were male. Characteristics of the study patients in each group are shown in Table 1. Six of 13 dogs (46.2%) that were classified as having a protein losing enteropathy based on a panhypoproteinemia noted on serum biochemistry, and 7/13 (53.8%) had normal protein levels on admission. All dogs without a protein losing enteropathy (n = 7) had received at minimum a 2 week hypoallergenic or novel protein dietary trial prior to enrollment with no significant response. Patients with a protein losing enteropathy had not received a dietary trial prior to study enrollment due to concern this would be insufficient treatment by itself in these patients, and due to concern in delaying further treatment given the critical nature of their condition. 3/7 dogs in the FMT group and 2/6 dogs in the placebo group had an upper and lower gastrointestinal endoscopy, whereas the remaining dogs had only an upper gastrointestinal endoscopy. Upper and lower gastrointestinal endoscopy was not performed in all patients due to concerns with a more prolonged anesthesia in patients with hypoalbuminemia. Histologic lesions in all patients were consistent with a diagnosis of IBD.

**Table 1:** Characteristics of dogs in the FMT and placebo groups at baseline.

Characteristic	FMT group	Placebo group
Number of females	71.4% (5/7)	83.3% (5/6)
Number of dogs receiving steroids prior to enrolment	57.1% (4/7)	50.0% (3/6)
Number of dogs receiving antimicrobials prior to enrolment	14.3% (1/7)	16.7% (1/6)
Number of dogs receiving probiotics prior to enrolment	42.9% (4/7)	16.7% (1/6)
Mean weight (kg)	20.0 +/-11.94	21.9 +/- 12.82
Mean age (years)	5.9 +/- 2.91	5.8 +/- 4.31
Mean CCECAI at baseline	6.32 +/-1.33	5.75 +/-1.44

Frequency and prevalence are reported for female sex and number of dogs receiving steroids, antimicrobials and probiotics. Means +/- standard error are reported for weight, age and CCECAI.

Abbreviations: FMT: Fecal microbial transplant, CCECAI: Canine chronic enteropathy clinical activity index.

### Treatment

The median dosage of prednisone received was 2.02mg/kg/day (range 1.01–2.31) amongst all dogs (Table 2). One dog in each treatment group had been initiated on an antimicrobial within 3 weeks of enrolment (one dog received a 1-week course of amoxicillin/clavulanic acid discontinued 1 week prior to enrolment, and one dog received a 1-week course of metronidazole discontinued 1-week prior to enrolment). Diets used during the study included Purina Proplan Veterinary Diets HA Hydrolyzed Dry Canine Formula (Purina, Missouri, United States of America) (n = 4), Royal Canin Hydrolyzed Protein Moderate Calorie Dry Canine Formula (Royal Canin, Aimargues, France) (n = 6), Rayne Clinical Nutrition Low Fat Kangaroo Maintenance Dry Canine Formula (Rayne Clinical Nutrition, Delaware, United States of America) (n = 1), and a novel protein diet formulated by the OVC HSC Clinical Nutrition Service (n = 2).

**Table 2:** Characteristics of included dogs.

Dog	Group	Age (years)	Sex	Breed	Prednisone dosage at time of treatment (mg/kg)	Diet	CCECAI		
							Day 0	Day 7	Day 30
1	FMT	10	FI	Mixed breed	2.2	Royal Canin HP	9	7	1
2	FMT	5	FS	Mixed breed	2.0	Rayne Kangaroo Low Fat	4	3	2.5
3	FMT	4	MI	German shepherd	1.4	Purina HA	7	7	3
4	FMT	6	FS	Yorkshire terrier	2.17	Royal Canin HP	3	1	0
5	FMT	8	FS	German shepherd	2.31	Royal Canin HP	12	5.5	3
6	FMT	1	FS	Labrador retriever	2.01	Purina HA	3	3	3
7	FMT	7	MN	Mixed breed	2.1	Royal Canin HP	5.5	5	.
8	Placebo	10	FS	German shepherd	1.1	Purina HA	5	1	1
9	Placebo	11	FS	Yorkshire terrier	2.0	Royal Canin HP	4	8	1
10	Placebo	7	MN	Pug	2.14	Purina HA	12	8	3
11	Placebo	5	FS	Mixed breed	2.2	Purina HA	8	4.5	5
12	Placebo	1	FS	Mixed breed	1.96	Home cooked diet formulated by OVC Nutrition Service	2.5	7	10
13	Placebo	1	FS	Mixed breed	1.96	Home cooked diet formulated by OVC Nutrition Service	3	7	9

Abbreviations: FMT: Fecal microbial transplant, Royal Canin HP: Royal Canin Hydrolyzed Protein Moderate Calorie Dry Canine Formula, Purina HA: Purina Proplan Veterinary Diets HA Hydrolyzed Dry Canine Formula, Rayne Kangaroo Low Fat: Rayne Clinical Nutrition Low Fat Kangaroo Maintenance Dry Canine Formula.

All 7 patients in the FMT group were available for reassessment at 7 days, and 6/7 were available at 30 days. All 6 patients in the placebo group were available for reassessment at 7 and 30 days. Three dogs received FMT at 30 days, and an additional 2 dogs received FMT at 90 days after enrolment due to incomplete treatment response at these times and were included in the FMT2 group. In total, 5/6 patients in the placebo group subsequently received FMT treatment due to insufficient response at 1 month (3 dogs) or at 3 months (2 dogs) following enrolment. No adverse effects were reported after FMT. One dog was euthanized within 1 month following enrolment due to suspected osteosarcoma development of the right forelimb and was thereby unavailable for further follow-up.

### Serum biomarkers

**Fig 1:**
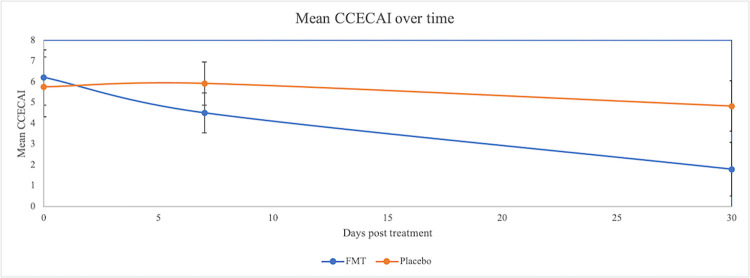
Mean CCECAI over time between the placebo and FMT group. No significant differences were noted between groups at each time point, although time remained a significant factor, with the lowest scores achieved by day 30 (p = 0.01).

**Fig 2:**
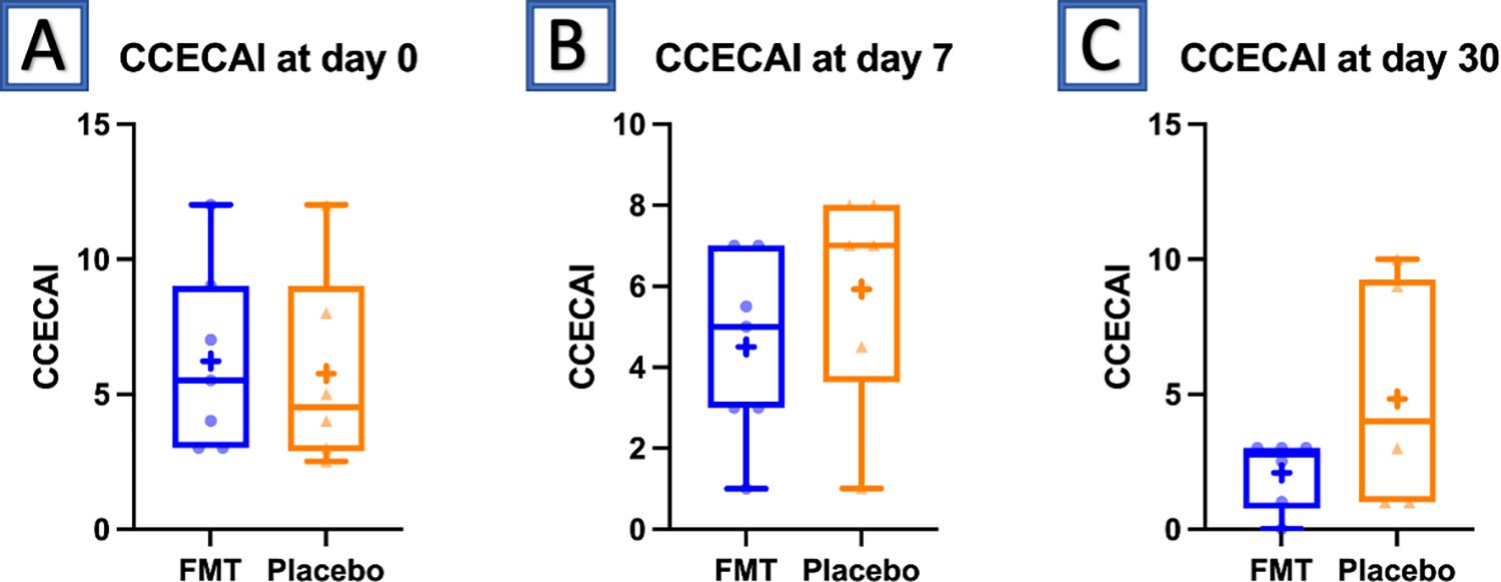
Box and whisker plot of the CCECAI at baseline, day 7 and day 30 in dogs that received placebo or FMT.

The CCECAI scoring throughout the study is shown in Table 3. The mean CCECAI at baseline, day 7, and day 30 is illustrated in Figs 1 and 2. In the dogs that received FMT, the mean CCECAI at baseline, day 7 and day 30 were 6.21 (+/- 1.33), 4.50 (+/- 0.97), and 1.78 (+/- 1.30) respectively. In dogs that received placebo, the mean CCECAI was 5.75 (+/- 1.44) at baseline, 5.92 (+/- 1.04) at day 7, and 4.83 (+/- 1.22) at day 30.

**Table 3:** The CCECAI between treatment groups at baseline, day 7 and day 30.

	Baseline	Day 7	Day 30
**Placebo**	5.75 +/- 1.44	5.92 +/- 1.04	4.83 +/- 1.22
**FMT**	6.21 +/- 1.33	4.50 +/- 0.97	1.78 +/- 1.30
**p-value**	0.82	0.34	0.12

No significant differences were noted between groups at each time point. Values for CCECAI are reported as means +/- standard errors.

Abbreviations: FMT: Fecal microbial transplant.

In 6/7 dogs that received FMT, the CCECAI at day 30 was lower than at baseline, and in no dog that received FMT did it worsen over time. Additionally, in all dogs that received FMT (n = 7), the CCECAI at day 30 was 3 or less (considered insignificant disease) [20]. In 4/6 dogs that received placebo treatment, the CCECAI score at day 30 was lower than at day 0. However, in two dogs that received placebo, the CCECAI steadily increased from day 0 to 30, and in one additional patient it worsened from day 7 to day 30. In 3/6 dogs that received placebo, the CCECAI at day 30 was 3 or less.

There were no differences between the FMT and placebo group at each time point (F test from ANOVA p = 0.40). The mean CCECAI in the placebo group (5.75) did not differ from that of the FMT group (6.32) at baseline (p = 0.82) or at day 30 (p = 0.12). However, there were trends for the FMT group to have a significantly lower CCECAI (1.78) at 30 days compared to baseline (p = 0.02). The mean CCECAI of the placebo group (4.83) at 30 days was not significantly different from baseline (p = 0.61). Time remained a significant factor for CCECAI, with the lowest scores achieved by day 30 (p = 0.01).

### The fecal microbiota

A total of 9753672 good quality reads were utilized for final analysis (median 180484/sample, range 129748–276776). Based on the sample with the smallest number of reads, a subsample of 129748 reads was used.

**Fig 3:**
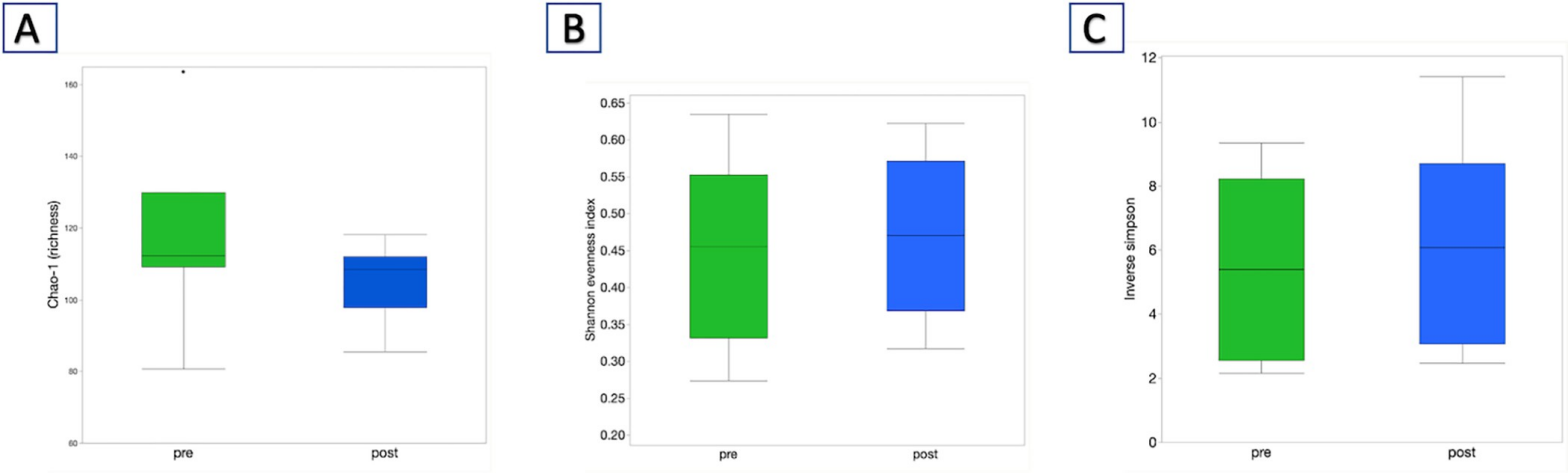
A) Chao-1, B) Shannon Evenness, and C) Inverse Simpson indices between the pre and one-week post FMT samples.

When comparing the microbiota at baseline compared to one week post FMT in patients that received FMT, there were no significant differences in alpha diversity between baseline and one-week post FMT samples for Chao (Fig 3A) Shannon Evenness (Fig 3B), and Inverse Simpson indices (Fig 3C), (p > 0.05 for all comparisons; p = 0.13 for Chao-1, p = 0.79 for Inverse Simpson, p = 0.61 for Shannon Evenness).

**Fig 4:**
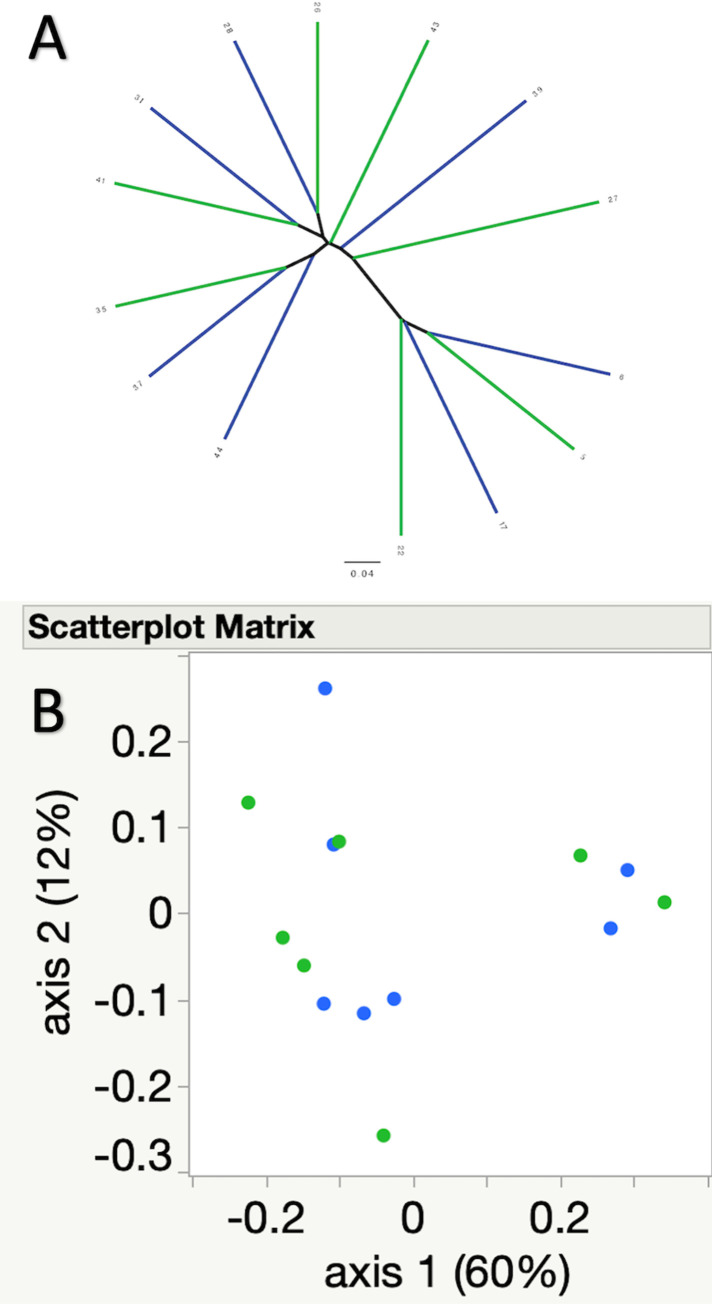
A) Dendrogram and B) principal coordinate analysis illustrating similarities and differences in community composition (Jaccard index) between the pre (green) and one-week post FMT (blue) samples. No significant differences were noted between pre and post FMT samples.

**Fig 5:**
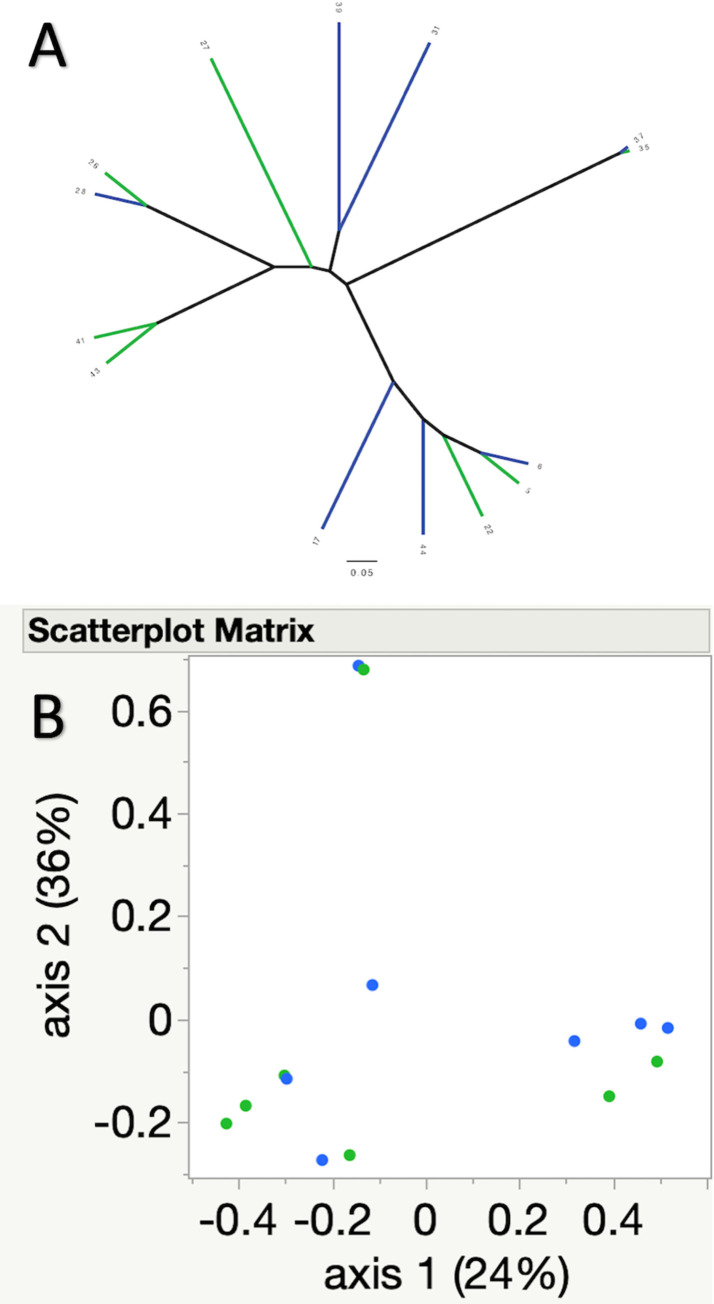
A) Dendrogram and B) principal coordinate analysis illustrating similarities and differences in community structure (Yue and Clayton index) between the pre (green) and one-week post FMT (blue) samples. No significant differences were noted between pre and post FMT samples.

Community membership (Jaccard index, p = 0.79), and structure (Yue and Clayton, p = 0.53) were not significantly different between pre and one-week post FMT samples. This is further illustrated by the lack of clustering of samples in the associated principal coordinate analyses (PCoA) and dendrograms (Fig 4 for Jaccard index, Fig 5 for Yue and Clayton index).

### Relative abundance and LefSe analysis

**Fig 6:**
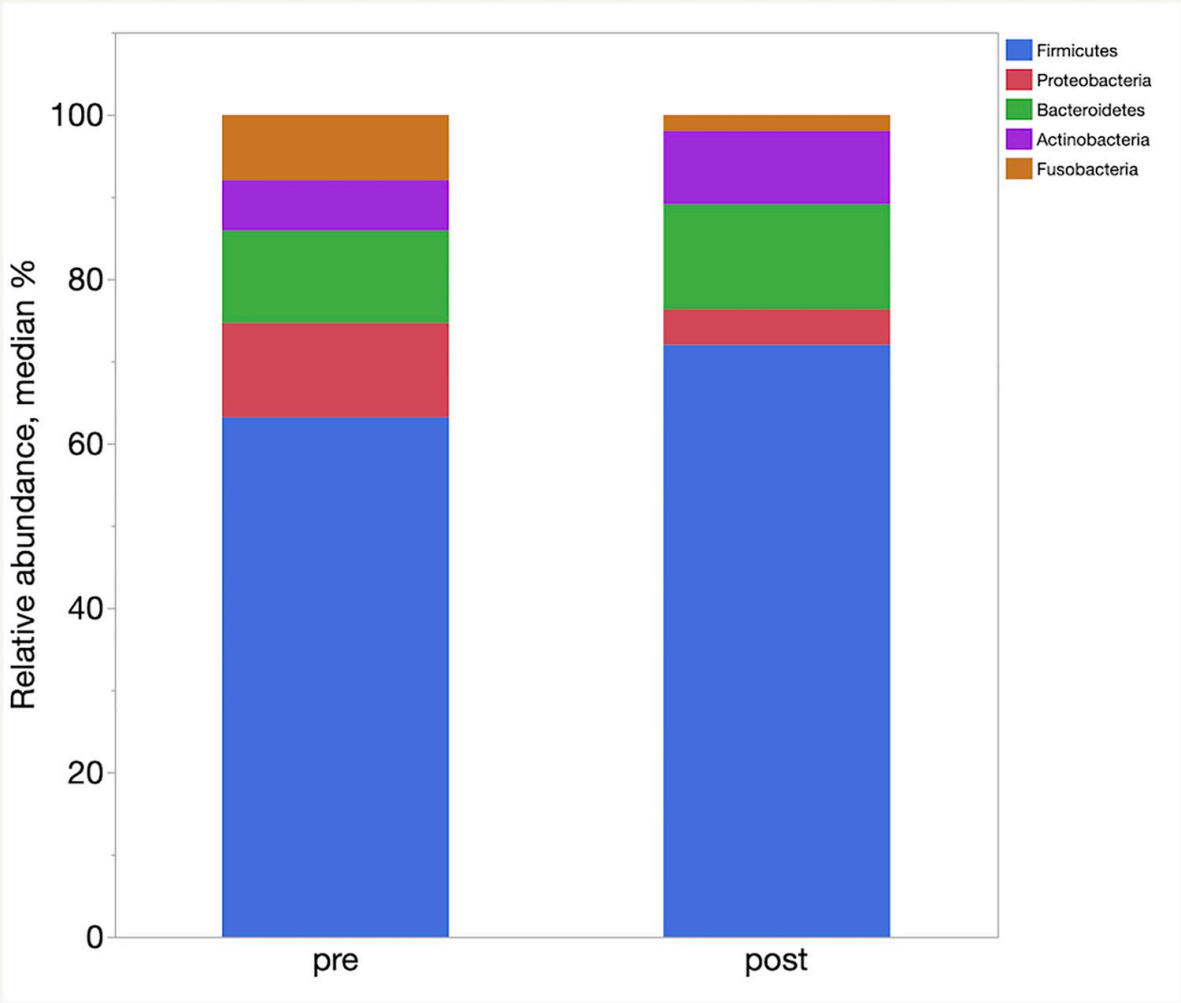
Relative abundances of the main bacterial phyla enriched between the pre and one-week post FMT samples.

**Fig 7:**
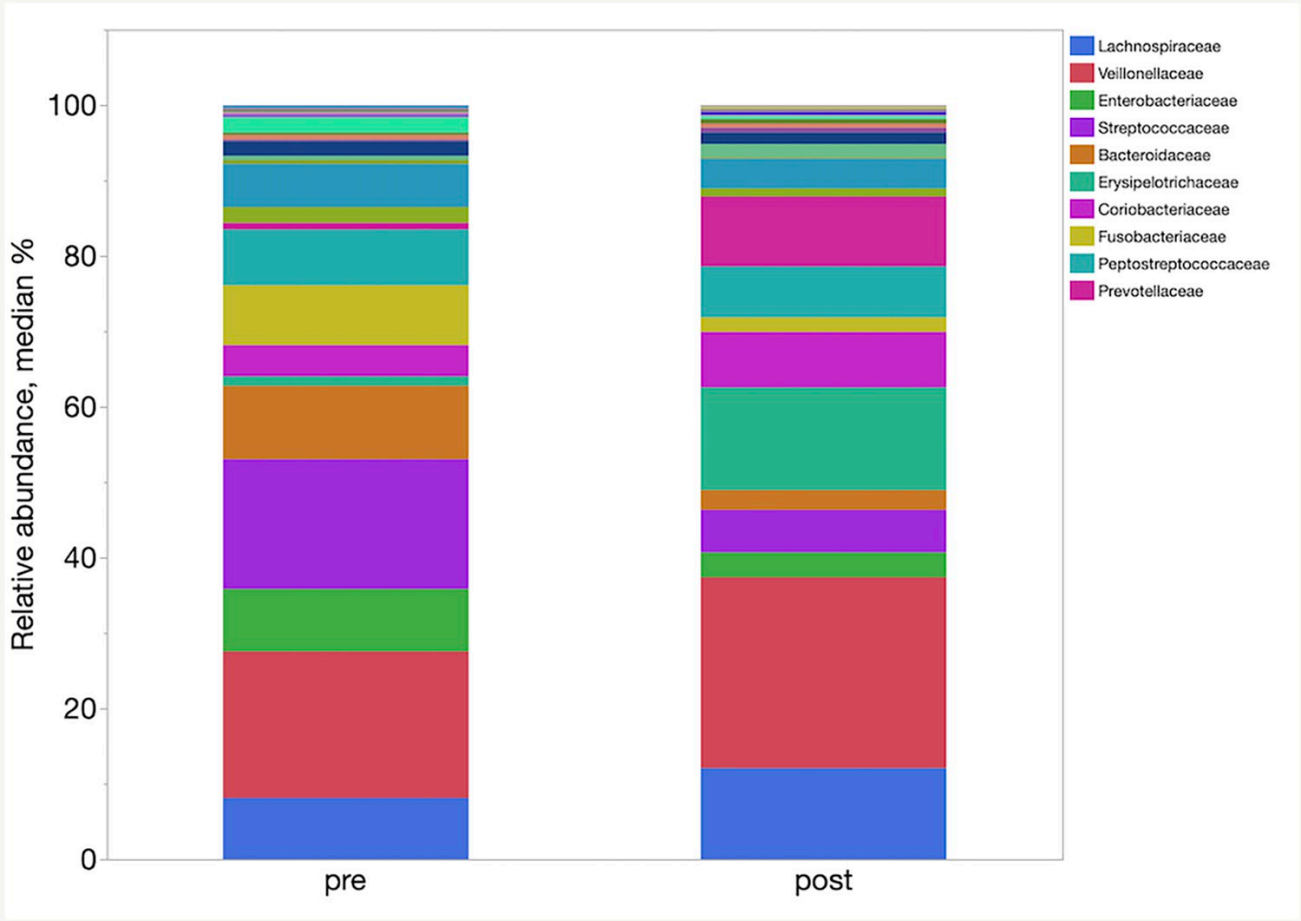
Relative abundances of the main bacterial families enriched between the pre and one-week post FMT samples.

**Fig 8:**
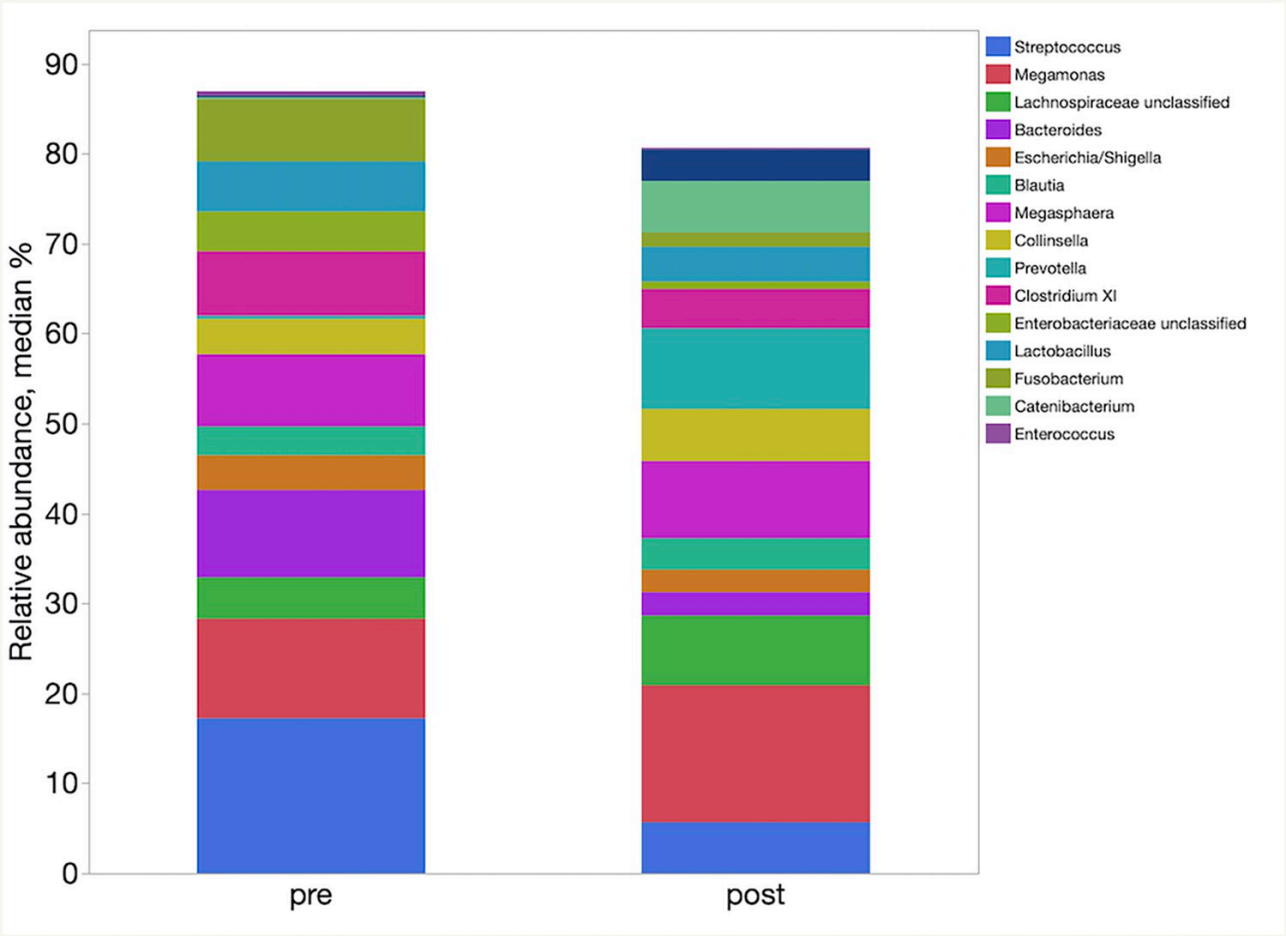
Relative abundances of the main bacterial genera enriched between the pre and one-week post FMT samples.

LefSe analysis demonstrated enrichment of different taxa in the different groups. Table 4 represents the main taxa (LDA >2) significantly associated with the post FMT samples. In LefSe analysis, the post FMT samples were enriched in families Ruminococcaceae, Coriobacteriaceae, and Erysipelotrichaceae, and genera *Faecalibacterium* and *Slackia*. The relative abundances of phyla, families and genera in pre and one-week post FMT samples are shown in Fig 6–8 respectively.

**Table 4:** LefSe of bacterial taxa and their association with the one-week post FMT samples, demonstrating taxa with an LDA >2.

LDA	pValue					
2.3	0.047	Firmicutes	Clostridia	Clostridiales	Ruminococcaceae	*Faecalibacterium*
2.9	0.006	Actinobacteria	Actinobacteria	Coriobacteriales	Coriobacteriaceae	*Slackia*
2.2	0.040	Firmicutes	Erysipelotrichia	Erysipelotrichales	Erysipelotrichaceae	Unclassified
2.4	0.012	Firmicutes	Bacilli	Bacillales	Unclassified	Unclassified

Abbreviations: LDA: Linear discriminant analysis.

## Discussion

Addition of FMT to standard treatment was feasible in this preliminary study. Subjectively, uptake in study enrolment was greater when FMT or placebo was offered at the time of endoscopy in dogs suspected to have IBD, compared to treatment following confirmation of histologic results consistent with IBD. Additionally, in this double-blinded trial, unblinding the treatment group after 1 month versus 3 months was subjectively more acceptable to owners and clinicians as this led to earlier additional intervention if clinical signs were poorly controlled with initial therapy. The information gained from this preliminary study will help refine methods for larger scale clinical trials and potentially increase likelihood of a successful trial.

In this preliminary study, there was no significant differences in the CCECAI at all time points between dogs in the placebo and FMT group. However, there were trends for dogs receiving FMT to have reductions in their disease severity, assessed by the CCECAI at day 30 compared to baseline, whereas dogs receiving placebo did not. However, power was limited at the interaction level, and therefore studies with greater power are necessitated to better determine whether FMT aids in faster response times compared to standard treatment alone. A clinical trial in puppies with parvovirus infection showed more rapid resolution of diarrhea and shorter hospitalization time for puppies receiving FMT [18]. Previous reports have also documented improved clinical scoring indices and fecal consistency of dogs with chronic enteropathy or IBD after FMT [28–30], as well as in dogs with IBD described as refractory to other treatments [31]. Potential benefits following FMT could include restoration of secondary bile acids, increased proportion of short chain fatty acids (SCFA) and SCFA producing bacteria, and resolution of dysbiosis [32–34].

The longer term effect on clinical scoring indices (such as the CCECAI) and remission rates following FMT in dogs with IBD remains unknown. However, FMT was found to be an easily applicable treatment option in this study. Therefore, this preliminary study demonstrated that studies investigating the benefit of FMT over a longer time frame with a larger sample size are warranted and that this is an area worthy of further exploration. Additionally, the response rate and other data from this study can be used to inform more precise sample size calculations for future investigations of FMT in dogs with IBD.

The addition of FMT to standard therapy of IBD did not significantly change fecal microbial diversity in recipients in this study. This contrasts with other reports of FMT in veterinary medicine. In a case series of FMT in 9 dogs with IBD, the fecal microbiota 2 weeks following FMT was more diverse with increased proportion of Fusobacteria [28]. Another case report of a dog with IBD showed that the fecal microbiota resembled that of the healthy, FMT donor dog’s microbiome at 2–14 days following FMT [30]. Although a single administration of FMT was performed in these case reports and case studies, it is possible the single FMT administration in the present study of dogs with IBD was insufficient to result in significant changes in alpha and beta diversity, and that the more intensive FMT administration protocols used in many human investigations are necessary to achieve microbial diversity in the recipient in some patients. Additionally, given the small sample size, a type II error as a cause for the lack of significant differences in diversity following FMT cannot be excluded.

Although measures of overall diversity did not significantly change following FMT in the present study, fecal samples 1-week after FMT were significantly enriched in Firmicutes, particularly family Ruminococcaceae and genus *Faecalibacterium* compared to baseline samples. Some members of Firmicutes, particularly members of Ruminococcaceae, and genus *Faecalibacterium* are essential SCFA-producing bacteria, and SCFA are proposed to have numerous beneficial and anti-inflammatory effects in the gastrointestinal tract [35,36]. Thereby, restoration of these essential SCFA-producing bacteria could be a potential benefit of FMT administration.

There are limitations to the present study. As a preliminary study, the sample size is small and limited the power and therefore the safety and efficacy conclusions that can be drawn, particularly at the interaction level. However, this study helped evaluate feasibility, acceptability, and uptake of FMT and will inform methodology for a larger clinical to make more robust conclusions regarding the longer term efficacy and safety of FMT in canine patients with IBD. Given that only 13 dogs were enrolled in a 2-year period in the present trial, multicentre trials are encouraged to help recruit a larger sample size for future larger-scale studies of FMT efficacy and safety. Additionally, a significant proportion of patients in the present study were panhypoproteinemic, and it is possible patients with IBD and panhypoproteinemia have a different response to FMT than patients without panhypoproteinemia. Adjunctive treatments (such as antacids, probiotics, or thromboprophylaxis) were not standardized, and thereby could have impacted the microbiota and influenced results or treatment outcome [37,38]. Future studies would benefit from increased power through a larger sample size, as well as randomization for adjunctive treatments (including probiotics) reducing confounding and variation.

Additionally, it is possible that the single administration of FMT in dogs with IBD in this study could be insufficient to result in changes to the microbiota where ongoing intestinal inflammation could perpetuate continued dysbiosis. A recent meta-analysis in people showed a higher clinical remission rate in people that received 10 or more FMT infusions in people with ulcerative colitis [39]. As well, in a review of FMT in canine patients, addition of glycerol was advised for frozen FMT preparations to reduce damage of microbial cells induced by freezing, although this was not performed in this investigation and potentially could have impacted the viability of frozen preparations [32]. Additionally, the fecal slurry used in this study was generated based on weight rather than bacterial potency. However, a recent review of FMT in veterinary medicine recommended FMT dosing based on fecal weight, and bacterial potency does not account for other potentially essential parts of the FMT, such as viruses, archaea and metabolites [32,40]. As well, in this study, microbial viability or composition of donor preparations was not assessed, and thereby could have impacted the efficacy of FMT should microbial viability have been sub-optimal. Thereby, it is possible the method of FMT preparation and administration used was inadequate to result in significant differences to the gastrointestinal microbiota in dogs with IBD in this study. Furthermore, the longer term impact of FMT in dogs with IBD was not investigated but should be assessed in future studies of FMT in dogs with IBD.

In conclusion this preliminary study was able to identify that addition of FMT is feasible and was well tolerated in this group of dogs 30 days after administration. Dogs receiving FMT in addition to standardized therapy did not have significant differences in the CCECAI at day 30 compared to the placebo group. Exploration of the utility of FMT for more rapid clinical improvement in IBD is warranted in future investigations of greater power, and over a longer time frame. In addition, further studies are required to assess the efficacy, safety, optimal FMT donor/patient characteristics, and utility of FMT in a larger group of patients, as well as comparing different FMT administration methods and protocols.

## Supporting information

S1 Table Patient data throughout the study. (XLSX)

S1 Appendix Initial patient data and diagnostic results. (DOCX)

## References

[1] Makielski K, Cullen J, O’Connor A, Jergens AE. Narrative review of therapies for chronic enteropathies in dogs and cats. J Vet Intern Med. 2019;33(1):11–22. doi: 10.1111/jvim.15345 30523666 PMC6335544

[2] Jergens A, Moore F, Haynes J, Miles K. Idiopathic inflammatory bowel disease in dogs and cats: 84 cases (1987–1990). J Am Vet Med Assoc [Internet]. 1992;201(10):1603–8. Available from: https://pubmed.ncbi.nlm.nih.gov/1289345/. 1289345

[3] Bernstein CN, Forbes JD. Gut Microbiome in Inflammatory Bowel Disease and Other Chronic Immune-Mediated Inflammatory Diseases. Inflamm Intestinal Dis. 2017;2(2):116–23. doi: 10.1159/000481401 30018962 PMC5988152

[4] DeGruttola AK, Low D, Mizoguchi A, Mizoguchi E. Current Understanding of Dysbiosis in Disease in Human and Animal Models. Inflamm Bowel Dis. 2016;22(5):1137–50. doi: 10.1097/MIB.0000000000000750 27070911 PMC4838534

[5] Atherly T, Rossi G, White R, Seo YJ, Wang C, Ackermann M, et al. Glucocorticoid and dietary effects on mucosal microbiota in canine inflammatory bowel disease. Plos One. 2019;14(12):e0226780. doi: 10.1371/journal.pone.0226780 31887117 PMC6936794

[6] AlShawaqfeh M, Wajid B, Minamoto Y, Markel M, Lidbury J, Steiner J, et al. A dysbiosis index to assess microbial changes in fecal samples of dogs with chronic inflammatory enteropathy. Fems Microbiol Ecol. 2017;93(11). doi: 10.1093/femsec/fix136 29040443

[7] Brandt LJ, Aroniadis OC. An overview of fecal microbiota transplantation: techniques, indications, and outcomes. Gastrointest Endosc. 2013;78(2):240–9. doi: 10.1016/j.gie.2013.03.1329 23642791

[8] Cammarota G, Ianiro G, Tilg H, Rajilić-Stojanović M, Kump P, Satokari R, et al. European consensus conference on faecal microbiota transplantation in clinical practice. Gut. 2017;66(4):569. doi: 10.1136/gutjnl-2016-313017 28087657 PMC5529972

[9] Costello SP, Hughes PA, Waters O, Bryant RV, Vincent AD, Blatchford P, et al. Effect of Fecal Microbiota Transplantation on 8-Week Remission in Patients With Ulcerative Colitis: A Randomized Clinical Trial. Jama. 2019;321(2):156. doi: 10.1001/jama.2018.20046 30644982 PMC6439766

[10] Suskind DL, Brittnacher MJ, Wahbeh G, Shaffer ML, Hayden HS, Qin X, et al. Fecal Microbial Transplant Effect on Clinical Outcomes and Fecal Microbiome in Active Crohn’s Disease. Inflamm Bowel Dis. 2015;21(3):556–63. doi: 10.1097/MIB.0000000000000307 25647155 PMC4329080

[11] Lee CH, Steiner T, Petrof EO, Smieja M, Roscoe D, Nematallah A, et al. Frozen vs Fresh Fecal Microbiota Transplantation and Clinical Resolution of Diarrhea in Patients With Recurrent Clostridium difficile Infection: A Randomized Clinical Trial. Jama. 2016;315(2):142–9. doi: 10.1001/jama.2015.18098 26757463

[12] Bakken JS, Borody T, Brandt LJ, Brill JV, Demarco DC, Franzos MA, et al. Treating Clostridium difficile infection with fecal microbiota transplantation. Clin Gastroenterology Hepatology Official Clin Pract J Am Gastroenterological Assoc. 2011;9(12):1044–9. doi: 10.1016/j.cgh.2011.08.014 21871249 PMC3223289

[13] Brandt LJ, Aroniadis OC, Mellow M, Kanatzar A, Kelly C, Park T, et al. Long-Term Follow-Up of Colonoscopic Fecal Microbiota Transplant for Recurrent Clostridium difficile Infection. Am J Gastroenterol. 2012;107(7):1079–87. doi: 10.1038/ajg.2012.60 22450732

[14] Cammarota G, Ianiro G, Gasbarrini A. Fecal Microbiota Transplantation for the Treatment of Clostridium difficile Infection. J Clin Gastroenterol. 2014;48(8):693–702. 24440934 10.1097/MCG.0000000000000046

[15] Moayyedi P, Surette MG, Kim PT, Libertucci J, Wolfe M, Onischi C, et al. Fecal Microbiota Transplantation Induces Remission in Patients With Active Ulcerative Colitis in a Randomized Controlled Trial. Gastroenterology. 2015 Jul 1;149(1):102–109.e6. doi: 10.1053/j.gastro.2015.04.001 25857665

[16] Paramsothy S, Kamm MA, Kaakoush NO, Walsh AJ, Bogaerde J van den, Samuel D, et al. Multidonor intensive faecal microbiota transplantation for active ulcerative colitis: a randomised placebo-controlled trial. The Lancet. 2017 Mar 25;389(10075):1218–28. doi: 10.1016/S0140-6736(17)30182-4 28214091

[17] Paramsothy S, Paramsothy R, Crohn’s DRJ of. Faecal microbiota transplantation for inflammatory bowel disease: a systematic review and meta-analysis. J Crohns Colitis. 2017;10(11):1180–99. 10.1093/ecco-jcc/jjx063 28486648

[18] Pereira GQ, Gomes LA, Santos IS, Alfieri AF, Weese JS, Costa MC. Fecal microbiota transplantation in puppies with canine parvovirus infection. J Vet Intern Med. 2018 Mar 1;32(2):707–11. doi: 10.1111/jvim.15072 29460302 PMC5867004

[19] Murphy T, Chaitman J, Internal EHJ of V. Use of fecal transplant in eight dogs with refractory Clostridium perfringens associated diarrhea [Abstract]. J Vet Intern Med. 2014;976–1134.

[20] Allenspach K, Wieland B, Gröne A, Gaschen F. Chronic Enteropathies in Dogs: Evaluation of Risk Factors for Negative Outcome. J Vet Intern Med. 2007 Jul 1;21(4):700–8. doi: 10.1892/0891-6640(2007)21[700:ceideo]2.0.co;2 17708389

[21] Klindworth A, Pruesse E, Schweer T, Peplies J, Quast C, Horn M, et al. Evaluation of general 16S ribosomal RNA gene PCR primers for classical and next-generation sequencing-based diversity studies. Nucleic Acids Res. 2013;41(1):e1–e1. doi: 10.1093/nar/gks808 22933715 PMC3592464

[22] Desjardins P, Conklin D. NanoDrop Microvolume Quantitation of Nucleic Acids. J Vis Exp. 2010;75(1):7537–41. doi: 10.3791/2565 21189466 PMC3346308

[23] Schloss PD, Westcott SL, Ryabin T, Hall JR, Hartmann M, Hollister EB, et al. Introducing mothur: open-source, platform-independent, community-supported software for describing and comparing microbial communities. Appl Environ Microbiol. 2009;75(23):7637–41. doi: 10.1128/AEM.01541-09 19801464 PMC2786419

[24] Arroyo LG, Rossi L, Santos BP, Gomez DE, Surette MG, Costa MC. Luminal and Mucosal Microbiota of the Cecum and Large Colon of Healthy and Diarrheic Horses. Animals. 2020;10(8):1403. doi: 10.3390/ani10081403 32806591 PMC7460328

[25] Quast C, Pruesse E, Yilmaz P, Gerken J, Schweer T, Yarza P, et al. The SILVA ribosomal RNA gene database project: improved data processing and web-based tools. Nucleic Acids Res. 2013;41(D1):D590–6. doi: 10.1093/nar/gks1219 23193283 PMC3531112

[26] Edgar RC, Haas BJ, Clemente JC, Quince C, Knight R. UCHIME improves sensitivity and speed of chimera detection. Bioinformatics. 2011;27(16):2194–200. doi: 10.1093/bioinformatics/btr381 21700674 PMC3150044

[27] Cole JR, Wang Q, Fish JA, Chai B, McGarrell DM, Sun Y, et al. Ribosomal Database Project: data and tools for high throughput rRNA analysis. Nucleic Acids Res. 2014;42(Database issue):D633–42. doi: 10.1093/nar/gkt1244 24288368 PMC3965039

[28] Niina A, Kibe R, Suzuki R, Yuchi Y, Teshima T, Matsumoto H, et al. Fecal microbiota transplantation as a new treatment for canine inflammatory bowel disease. Biosci Microbiota Food Heal. 2021;40(2):98–104. doi: 10.12938/bmfh.2020-049 33996366 PMC8099633

[29] Niina A, Kibe R, Suzuki R, Yuchi Y, Teshima T, Matsumoto H, et al. Improvement in Clinical Symptoms and Fecal Microbiome After Fecal Microbiota Transplantation in a Dog with Inflammatory Bowel Disease. Vet Medicine Res Reports. 2019;10:197–201. 10.2147/VMRR.S230862 PMC6898721 31819862

[30] Weese J, Costa M, Webb J. Preliminary clinical and microbiome assessment of stool transplantation in the dog and cat. J Vet Intern Med. 2013;

[31] Bottero E, Benevenuti E, Ruggiero P. Trapianto del microbiota fecale (FMT) in 16 cani affetti da IBD idiopatica.

[32] Chaitman J, Gaschen F. Fecal Microbiota Transplantation in Dogs. Vet Clin North Am Small Animal Pract. 2020;51(1):219–33. 10.1016/j.cvsm.2020.09.012 33131919

[33] Khoruts A, Sadowsky MJ. Understanding the mechanisms of faecal microbiota transplantation. Nat Rev Gastroentero. 2016;13(9):508–16. doi: 10.1038/nrgastro.2016.98 27329806 PMC5909819

[34] Kelly CR, Kahn S, Kashyap P, Laine L, Rubin D, Atreja A, et al. Update on Fecal Microbiota Transplantation 2015: Indications, Methodologies, Mechanisms, and Outlook. Gastroenterology. 2015 Jul 1;149(1):223–37. doi: 10.1053/j.gastro.2015.05.008 25982290 PMC4755303

[35] Minamoto Y, Otoni CC, Steelman SM, Büyükleblebici O, Steiner JM, Jergens AE, et al. Alteration of the fecal microbiota and serum metabolite profiles in dogs with idiopathic inflammatory bowel disease. Gut Microbes. 2014 Dec 22;6(1):33–47. 10.1080/19490976.2014.997612 PMC4615558 25531678

[36] Minamoto Y, Minamoto T, Isaiah A, Sattasathuchana P, Buono A, Rangachari VR, et al. Fecal short-chain fatty acid concentrations and dysbiosis in dogs with chronic enteropathy. J Vet Intern Med. 2019;33(4):1608–18. doi: 10.1111/jvim.15520 31099928 PMC6639498

[37] Freedberg DE, Toussaint NC, Chen SP, Ratner AJ, Whittier S, Wang TC, et al. Proton Pump Inhibitors Alter Specific Taxa in the Human Gastrointestinal Microbiome: A Crossover Trial. Gastroenterology. 2015;149(4):883–885.e9. doi: 10.1053/j.gastro.2015.06.043 26164495 PMC4584196

[38] Garcia‐Mazcorro JF, Suchodolski JS, Jones KR, Clark‐Price SC, Dowd SE, Minamoto Y, et al. Effect of the proton pump inhibitor omeprazole on the gastrointestinal bacterial microbiota of healthy dogs. Fems Microbiol Ecol. 2012;80(3):624–36. doi: 10.1111/j.1574-6941.2012.01331.x 22324305

[39] Syal G, Kashani A, Shih DQ. Fecal Microbiota Transplantation in Inflammatory Bowel Disease- a Primer for the Internists. Am J Medicine. 2018;131(9):1017–24. 10.1016/j.amjmed.2018.03.010 29605414

[40] Mentula S, Harmoinen J, Heikkilä M, Westermarck E, Rautio M, Huovinen P, et al. Comparison between Cultured Small-Intestinal and Fecal Microbiotas in Beagle Dogs. Appl Environ Microb. 2005;71(8):4169–75. doi: 10.1128/AEM.71.8.4169-4175.2005 16085799 PMC1183360

