# Supplementary material for: Investigating fecal microbial transplant as a novel therapy in dogs with inflammatory bowel disease: A preliminary study
Source: PLoS One. 2022 Oct 18;17(10):e0276295. doi: 10.1371/journal.pone.0276295 (PMC9578606; doi:10.1371/journal.pone.0276295)
Supplement: S1 Appendix — (DOCX) [file pone.0276295.s002.docx]

**Appendix 1:** Initial patient data and diagnostic results

| **ID** | **Group** | **Age (years)** | **Sex** | **Total protein (g/L)** | **Albumin (g/L)** | **HCT (%)** | **WBC count (x10^9/L)** | **Histopathology (morphologic diagnosis)** | **Completed study?** |
| --- | --- | --- | --- | --- | --- | --- | --- | --- | --- |
| 652113 | FMT | 10 | FI | 40 | 24 | 53 | 11.0 | Small intestine: Chronic plasmacytic and lymphocytic enteritis  Stomach: chronic lymphoplasmacytic gastritis | Yes |
| 654114 | FMT | 5 | FS | 32 | 13 | 54 | 16.6 | Small intestine: Chronic lymphoplasmacytic to granulomatous enteritis with villous blunting, lymphangiectasia, crypt ectasia and hyperplasia  Stomach: chronic lymphoplasmacytic gastritis, with erosion and lymphoid hyperplasia  Colon: Chronic lymphoplasmacytic colitis with edema | Yes |
| 658521 | FMT | 4 | MI | 55 | 33 | 48 | 11.0 | Small intestine: Plasmacytic enteritis with cryptectasia  Stomach: Normal stomach | Yes |
| 659520 | FMT | 6 | FS | 34 | 14 | 67 | 18.3 | Small intestine: Chronic plasmacytic and lymphocytic enteritis, with cryptectasia  Stomach: chronic lymphoplasmacytic gastritis | Yes |
| 614882 | FMT | 8 | FS | 30 | 17 | 44 | 19.2 | Small intestine: Plasmacytic lymphocytic enteritis  Stomach: Plasmacytic and lymphocytic gastritis with edema | Yes |
| 663440 | FMT | 1 | FS | 53 | 29 | 45.8 | 6.72 | Small intestine: Lymphoplasmacytic eosinophilic enteritis with villous atrophy | Yes |
| 665759 | FMT | 7 | MN | 56 | 29 | 49 | 13.1 | Small intestine: Chronic lymphoplasmacytic enteritis  Stomach: chronic lymphoplasmacytic gastritis with lymphoid follicular hyperplasia  Colon: chronic erosive lymphoplasmacytic colitis with hemorrhage | No; euthanized 1 month after enrolment due to suspected osteosarcoma |
| 640898 | Placebo | 10 | FS | 32 | 17 |  |  | Small intestine: Chronic plasmacytic and lymphocytic enteritis, with crypt ectasia, hyperplasia  Stomach: Chronic lymphoplasmacytic gastritis  Colon: chronic lymphoplasmacytic colitis with edema | Yes |
| 653470 | Placebo | 11 | FS | 23 | 11 | 46 | 22.1 | Small intestine: Chronic lymphoplasmacytic and neutrophilic/erosive enteritis with moderate to marked crypt distension  Stomach: erosive and lymphocytic gastritis with edema | Yes |
| 655299 | Placebo | 7 | MN | 31 | 10 | 31 | 29.6 | Small intestine: Chronic lymphoplasmacytic enteritis with crypt distension  Stomach: Chronic lymphoplasmacytic gastritis | Yes |
| 654475 | Placebo | 5 | FS | 43 | 27 | 48 | 17.8 | Small intestine: Chronic lymphoplasmacytic and pyogranulomatous enteritis  Stomach: Chronic lymphocytic gastritis | Yes |
| 658263 | Placebo | 1 | FS | 57 | 33 | 52 | 8.4 | Small intestine: Chronic lymphoplasmacytic and neutrophilic enteritis  Stomach: chronic plasmacytic and lymphocytic gastritis | Yes |
| 660702 | Placebo | 1 | FS | 67 | 39 | 54 | 7.6 | Small intestine: Chronic plasmacytic and lymphocytic enteritis  Stomach: Chronic lymphoplasmacytic gastritis with edema  Colon: Chronic plasmacytic lymphocytic colitis with edema and hemorrhage | Yes |
